# Bone-inspired enhanced fracture toughness of de novo fiber reinforced composites

**DOI:** 10.1038/s41598-019-39030-7

**Published:** 2019-02-28

**Authors:** Flavia Libonati, Andre E. Vellwock, Francesco Ielmini, Dilmurat Abliz, Gerhard Ziegmann, Laura Vergani

**Affiliations:** 1 Department of Mechanical Engineering, Politecnico di Milano, via G. La Masa 1, 20156 Milano, Italy; 2 Department of Polymer Materials and Plastics Engineering, Clausthal University of Technology, Clausthal-Zellerfeld, Germany

## Abstract

Amplification in toughness and balance with stiffness and strength are fundamental characteristics of biological structural composites, and a long sought-after objective for engineering design. Nature achieves these properties through a combination of multiscale key features. Yet, emulating all these features into synthetic *de novo* materials is rather challenging. Here, we fine-tune manual lamination, to implement a newly designed bone-inspired structure into fiber-reinforced composites. An integrated approach, combining numerical simulations, ad hoc manufacturing techniques, and testing, yields a novel composite with enhanced fracture toughness and balance with stiffness and strength, offering an optimal lightweight material solution with better performance than conventional materials such as metals and alloys. The results also show how the new design significantly boosts the fracture toughness compared to a classic laminated composite, made of the same building blocks, also offering an optimal tradeoff with stiffness and strength. The predominant mechanism, responsible for the enhancement of fracture toughness in the new material, is the continuous deviation of the crack from a straight path, promoting large energy dissipation and preventing a catastrophic failure. The new insights resulting from this study can guide the design of *de novo* fiber-reinforced composites toward better mechanical performance to reach the level of synergy of their natural counterparts.

## Introduction

Structural components and materials continuously face damage during their lifetime. Defects, often caused by the manufacturing process or by accidental events, are unavoidable and may put the structures or materials in danger, causing risk to human safety. The main challenge for researchers is to improve the flaw tolerance of materials, increasing their safety, rather than preventing defects. Most conventional monolithic materials face a typical strength-toughness dichotomy: metals are well-known for the high toughness but reduced strength, whereas ceramics offer remarkable compressive strength but suffer from a limited toughness^1,2^. Laminated composites, also among the most common motifs in biological materials^3–5^, generally represent an optimal cost-effective solution for lightweight structural design, offering high specific stiffness and strength, but a limited toughness^6^. Some shortcomings of classic laminates, such as delamination and low impact resistance, still remain unsolved issues^6,7^. Yet, slight improvements have been shown by different techniques that are trying to face the 3D composite challenge, such as 3D-weaving^8^, the addition of vertically-aligned CNTs^9^, out-of-plane pins^10,11^ or fibers^12^. Also, fiber-reinforced polymers (FRPs), such as fiberglass (GFRP) or carbon fiber-reinforced polymers (CFRP), widely used in aerospace, automotive, civil infrastructure, and for sporting products, do not attain an amplification in the mechanical properties compared to their constituents. Thus, it is necessary to develop new design strategies to deliver future superior composites for such applications.

Amplification in toughness and balance with stiffness and strength are fundamental characteristics of biological structural composites, such as bone and nacre^13,14^, and a coveted objective for engineering design. Nature achieves these properties through a combination of key features (e.g. heterogeneity, nano-confinement of substructures, sophisticated interfaces, and hierarchy), resulting from a billion-year-long evolution^15^. Yet, mimicking all these features into the design and fabrication of *de novo* materials is complex and challenging, despite the recent progress in manufacturing^1,15^. Albeit these recurring motifs in natural materials^4,16–21^, large diversity has been achieved in Nature using a limited palette of universal meager constituents (i.e. minerals and proteins), aka building blocks^22^. Man instead, uses a wide range of monolithic materials to achieve a good variety of synthetic composites, also generating a lot of waste and rising the recycling issues^23^. Among natural materials, biomineralized tissues generally offer an optimal strength-toughness tradeoff. This unfolds their function as structural materials: seashells and nacre have developed a large impact resistance, offering protection from predators’ sharp-toothed attacks, whereas bone, continuously subjected to loading, provides large fracture toughness, damage tolerance, and the ability of self-repairing. Biominerals are all characterized by two main building blocks: a mineral, typically a hard calcium carbonate that confers stiffness and strength to the whole material, and an organic phase, which acts as a compliant matrix, allowing deformation and promoting energy-dissipating mechanisms. These are organized into a hierarchical composite configuration, which promotes the activation of different mechanisms at different length scales, resulting in an overall amplification in the mechanical properties, far beyond those described by the simple composite rule of mixture^14^.

Bone and nacre are the most-known hard tissues. Despite their structural similarities, the two biominerals seem to have adopted completely different strategies for achieving mechanical robustness^24^. Bone has a highly sophisticated hierarchical organization consisting of seven rather complex substructures. Nacre, instead, has a very simple layered structure, characterized by a brick-and-mortar pattern. The simplicity of nacre makes it largely adopted as a biomimetic model, hence extensively mimicked by several manufacturing techniques, from freeze casting to hot pressing and additive manufacturing^25–33^. To mimic bone, instead, one has to face the challenge of implementing different substructures with different levels of precision, which is a trait of each manufacturing technique. In bone, the substructure that provides the largest contribution to toughness enhancement is the microscale, where several extrinsic and intrinsic mechanisms are activated^34,35^. The most organized bone microstructural configuration, aka Haversian structure, has been extensively studied^36–39^. It has a composite configuration where repetitive tubular units, the osteons, are interspersed into a more mineralized and apparently less organized phase, called interstitial. The osteons are secondary structures, originating from the remodeling process: they are made of concentric lamellae, receive the primary nutrition from the central vascular canal, and are connected to the interstitial matrix through a weaker interface^40^, dense of microcracks^34^, named cement line, which plays a crucial role in deflecting cracks^35,41–43^. Despite the largest contribution to toughness increase has been ascribed to microscale mechanisms (typical of the Haversian structure), only a few authors have tried to mimic the microstructural features into new design^44–48^. This might be due to the fact that bone microstructure is more complex than the nacreous brick-and-mortar topology and replicating the tubular elements is rather challenging. 3D-printing still represents the most versatile and promising technique to implement such complex design. It is appropriate as proof-of-concept, to investigate the role of design features and to perform systematic studies. However, owing to some current material limitations, it falls short when it comes to fabricating a material for real applications, also providing a comparison with currently adopted structural materials.

The discovery of natural materials’ excellent performance has spurred the research in biomimicry, leading to the development of appealing solutions^20,32,49^. In particular, synthetic materials able to mimic such natural motifs could have a large impact on many engineering fields, especially energy-related and transportation industry. FRPs today represent the most adopted solution for lightweight structural components, in spite of their limitations. Here we show how to implement bone microstructural features and the corresponding toughening mechanisms into large-scale materials with potential lightweight structural applications. We ensure a low weight and achieve - for the first time - an enhanced fracture toughness compared to currently adopted structural materials. Drawing inspiration by the microstructure of cortical bone enables us to reproduce the crack deflection and twisting mechanisms, which are thought to be the main contributors to bone fracture resistance^34^, and to boost the fracture toughness with respect to the mostly used lightweight composites (e.g. laminates). Our design, guided by previous experimental results^45^ and numerical modeling^47^, has been adjusted to be manufactured by a custom-developed VARTM (vacuum assisted resin transfer molding) process. For comparative aims, a classic laminate has also been fabricated. Compared to conventional laminates, which typically fail by delamination, our bioinspired topology is expected to guide fracture along its tortuous interfaces. In addition, we present a 2D numerical model, based on XFEM (eXtended Finite Element Method), building on a previous simulation study^47^ focused on a former bioinspired design^45^. The numerical model is used: first to guide the design phase, then to unravel the role of structural features in the fracture process, and improve the final design. This work uses a comprehensive approach, combining simulations, manufacturing, and experiments to elucidate the role of characteristic Haversian features in bone’s enhanced fracture toughness. Additionally, it shows how to implement biologically-inspired motifs into the design of large-scale materials with multiple functions (e.g. weight reduction, body support, enhanced resistance to fracture and stiffness) and potential direct applications in industry.

## Results and Discussion

### Design process

**Figure 1:**
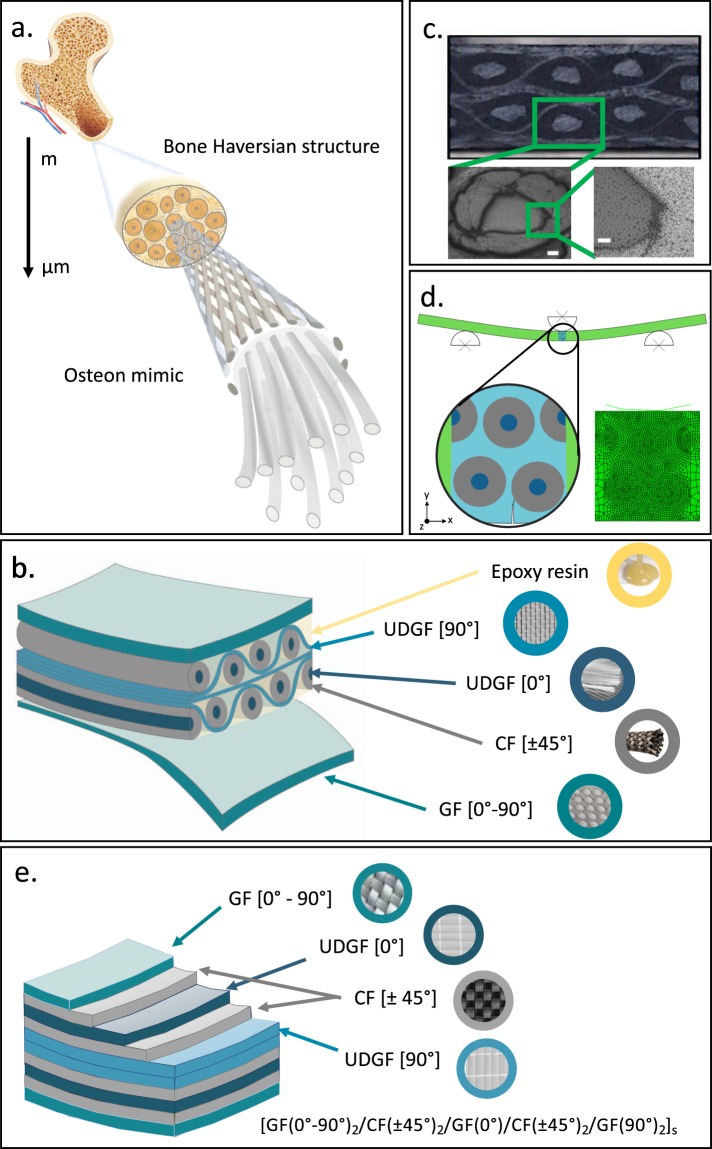
Nature inspiration, material design, and manufacturing. **(a)** Inspiration by the Haversian structure, characteristic of bone tissue at the microscale. Magnification of the main feature, the osteon, mimicked by a tube, made of ±45°-oriented carbon fibers, filled up with unidirectional glass-fibers. (**b)** Schematic of the biomimetic architecture, named Bio-2, and fundamental building blocks (carbon fibers, glass fibers, and epoxy resin). (**c)** Top picture showing the cross-section of the manufactured bioinspired composite material with magnifications, from SEM, of the osteon-like cross section. Bottom left picture (scale bar 200 μm) showing the osteon-like cross section. Bottom right picture (scale bar 50 μm) showing both the inner region, made of unidirectional glass-fibers, and the outer border, made of carbon fibers. (**d)** Finite element model of the transversal three-point bending case study, with a magnification showing the simplified topology and the finer mesh in the central region. (**e)** Schematic of the classic laminate architecture, Lam-2, including constituents and stacking sequence.

Fig. 1 shows the design process, from the biomimetic model to the final design and the manufactured material. The geometry mimics the osteonal secondary structure of mammalian bone, represented in the schematics of Fig. 1a. Osteons, cement sheaths, lamellae, and interstitial tissues have been implemented through carbon fibers (CF), glass fibers (GF), and epoxy matrix (Fig. 1b). Bundles of unidirectional UD-GF [0°], embedded into ±45°-CF sleeves (fabric type: Twill 2 × 2), mimic the osteons and the outer sheaths. The osteons are placed into a staggered configuration, with three layers of non-crimp fabric, made of UD-GF [90°], wrapped around to mimic an interconnected system as the interstitial one. The orientation of the UD-GF is orthogonal to the main osteon direction, providing a balance in the fiber orientation and also ensuring good performance of the whole material in the transversal direction. The outer circumferential system is mimicked by a bidirectional woven GF fabric (Twill 2 × 2) [0°–90°]. The whole system is impregnated by epoxy resin. Fig. 1c shows the cross-section of the manufactured material, from now on named Bio-2. Besides the above described design (Bio-2), we also propose another design solution (Bio-2-CNT), having the same structural topology of Bio-2 and the addition of CNTs to the epoxy matrix. The latter solution is aimed at delivering further toughness enhancement, by introducing CNT-driven small-scale toughening mechanisms. Here we expect the activation of multiscale toughening mechanisms: i) large-scale toughening mechanisms (i.e. crack deflection and splitting), fostered by the topological pattern, and ii) small-scale toughening mechanisms (i.e. micro-cracking), promoted by the nano-reinforcement, reaching a synergistic effect. Bio-2 represents an evolution of a former design (here called Bio-1), described in detail in a previous work^45^, where the osteons are mimicked by ±45°-CF sleeves filled up with longitudinal GF, aligned with bundles of UD-GF, enclosed by two UD-GF fabrics, and embedded into epoxy matrix.

Compared to the real bone architecture, Bio-2 has a similar osteon volume fraction (i.e. about 60%)^50,51^, but is one order of magnitude larger, being intended for large-scale structural applications. Owing to some intrinsic difficulties in the manufacturing process, the design has been simplified by neglecting some features, such as the Haversian canals and the canaliculi. Those features play an active role in bone remodeling. However, being our material synthetic, we believe that neglecting them will not have a detrimental effect on the overall material performance.

To investigate the effect of the bioinspired design and provide a direct comparison with currently adopted materials, we also fabricate a classic laminated composite with a layered architecture, named Lam-2, consisting of the same building blocks (GF, CF, epoxy) in the same amount and orientation. The schematic of the laminate is shown in Fig. 1e. All the materials, object of this study, are made of about 50% vol. fibers (50%-GF and 50%-CF). The Bio-2-CNT includes the addition of 0.1%wt. CNTs. Details regarding the material design are provided in Table S1, Supplementary Information.

### Ad hoc manufacturing

Lam-2 is fabricated by classic manual lamination. To fabricate the bioinspired composite plates instead, we develop a custom-developed VARTM process, described in detail in the Methods section. From the manufactured composite plates, we cut 90 samples, and carry out static testing.

### Experimental testing and simulations

The experimental campaign includes testing under different loading conditions (i.e. tensile, compressive, and flexural) and different directions (i.e. longitudinal and transversal with respect to the main osteon direction), allowing a direct comparison between the performance of the bioinspired designs (Bio-2 and Bio-2-CNT) and that of the classic laminate (Lam-2), also accounting for the anisotropy. We also perform translaminar fracture toughness tests to be able to experimentally determine the fracture performance of the various solutions and compare the outcome with conventional materials from the literature (e.g. metals and classic composites). The details of sample geometry and testing setup are included in the Methods section and Supplementary Information.

**Figure 2:**
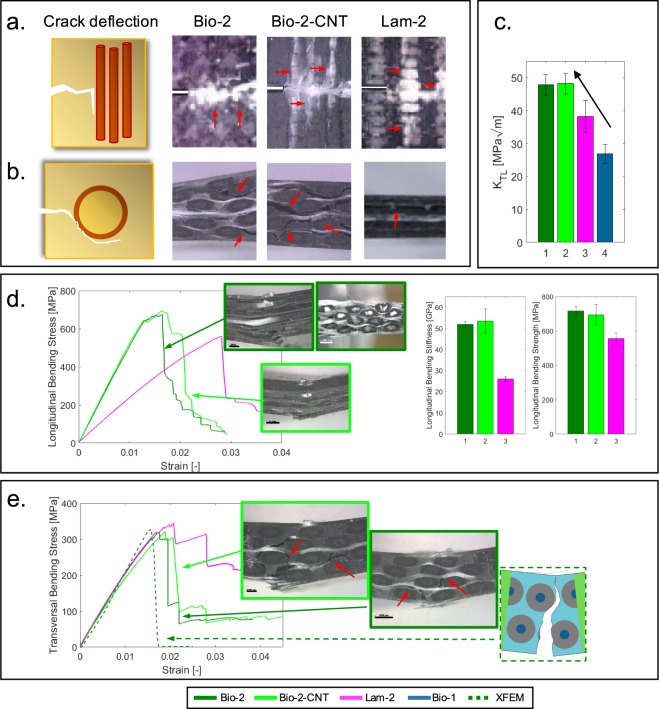
Experimental and numerical results. (**a)** Schematic of crack deflection in bone (longitudinal plane) and pictures (from optical microscope) of crack deflection in the composites. (**b)** Schematic of crack deflection in bone (transversal plane) and snapshots of crack deflection in the composites. The bioinspired composites (Bio-2 and Bio-2-CNT) reproduce the crack deflection around the osteon and the longitudinal splitting. (**c)** Bar plot showing the results of the translaminar fracture toughness tests: the new biomimetic designs (Bio-2 and Bio-2-CNT) boost the fracture toughness by 26% compared to the classic laminate (Lam-2) and by 86% compared to the previous design presented in^45^. (**d)** Results of the longitudinal three-point bending case study showing better performance of the biomimetic design, in terms of stiffness and strength (pictures from stereomicroscope, scale bar 1000 μm for top left figure and 2000 μm for the other two). (**e)** Results of the transversal three-point bending case study showing comparable properties of the biomimetic solutions and the classic laminate. The numerical model (dark green dashed line) shows good agreement with the experimental results (dark green continuous line) and is able to accurately predict the crack path. Experimental pictures from stereomicroscope (scale bar 1000 μm for the left figure and 2000 μm for the right one). Color legend at the bottom.

Fig. 2 displays the main outcome of the mechanical tests, focusing on specific loading case scenarios. The effect of the topology is evident from the pictures shown in Fig. 2. The bone-like architecture clearly influences the failure process, driving the crack through a complex path and allowing for a gradual energy release. The latter can also be revealed by stress-strain curves, where we first observe a load-drop, then a stepwise reduction, following the progressive damaging process. The crucial toughening mechanism is the crack deviation: when a main propagating crack encounters an osteon, it generally deviates its path from a straight line and follows the osteon curvature. Fig. 2a,b shows a direct comparison between the fundamental mechanisms of crack deviation, represented with a schematic, and the corresponding mechanisms observed during testing. In some cases, we also notice the crack branching, which allows for further energy dissipation. In the bioinspired designs we can observe the crack deviation in both longitudinal and transversal planes. This mechanism can be *in situ* observed, during transversal testing (Fig. 2e). For the longitudinal loading cases (Fig. 2d) instead, to investigate the effect of the geometrical features, it is essential to analyze the pictures of the failure surface, taken with a stereomicroscope (Zeiss Discovery V12). One limitation regards the possibility of observing only one plane. However, considering the final failure, we believe that out-of-plane crack deviations occurred, leading to twisting onto different planes. In the case of the classic laminate, Lam-2, the major failure mechanism is the delamination, occurring between different planes (Fig. 2b). Other minor mechanisms include fiber-matrix debonding and matrix cracking. Microscopic observations allow one to identify the failure mechanisms, confirming many similarities between the toughening mechanisms occurring in the bioinspired composites (Bio-2 and Bio-2-CNT) and those occurring in the microstructure of cortical bone (Fig. 2a,b). We can also notice how, despite the scale difference between our materials and bone microstructure, the main toughening mechanisms have been correctly mimicked, at a larger length scale. This is common to many other studies^20,26,30,33,52,53^ where, despite the scale difference, the authors could correctly capture the typical mechanisms noticed in the natural counterparts.

These topology-driven toughening mechanisms play a key role in the mechanics of the bioinspired materials, leading to a leap in the mechanical performance. In particular, if we observe the longitudinal flexural case study (Fig. 2d), which is also the most common loading condition for bone (e.g. femur)^54^, the bioinspired designs have a superior response, with a two-fold increase in stiffness and a 30% increase in strength, compared to the composite laminate (Lam-2).

In transversal direction, the bioinspired solutions also show comparable mechanical properties with respect to the laminate Lam-2, in spite of their topology-induced anisotropy (Fig. 2e) owing to the main osteon alignment. Indeed, the new biomimetic designs bring a large improvement in transversal properties with respect to a former design presented in^45^. We also simulate the flexural loading condition in transversal direction using XFEM, finding good agreement with the experimental results in terms of stress-strain trend (Fig. 2e). Additionally, the numerical model can accurately predict the phenomenon of crack propagation in the biomimetic design, also providing further information on the effect of the topological features on the overall fracture response.

The outcome of the experimental campaign, summarized in Tables S3 and S4, Supplementary Information, does not show a big effect of CNTs though, as the mechanical response of Bio-2 and Bio-CNT are generally comparable. The reason might be due to the small amount of CNT (0.1%wt.) added to the resin. However, the amount of the CNTs is also constrained by the manufacturing process. A larger amount of CNTs would have increased the viscosity of the resin, making the impregnation process difficult and rising the risk of manufacturing-induced defects.

The results of the translaminar fracture toughness tests, summarized in the bar plot in Fig. 2c, show a large leap in fracture toughness of the new proposed bioinspired composites (Bio-2 and Bio-2-CNT) compared to the former design (Bio-1)^45^, by 86%, and compared to the classic laminate (Lam-2), by 26%. For the bio-inspired topologies, we perform the translaminar fracture toughness tests in longitudinal direction (i.e. applied load parallel to the main osteon direction).

**Figure 3:**
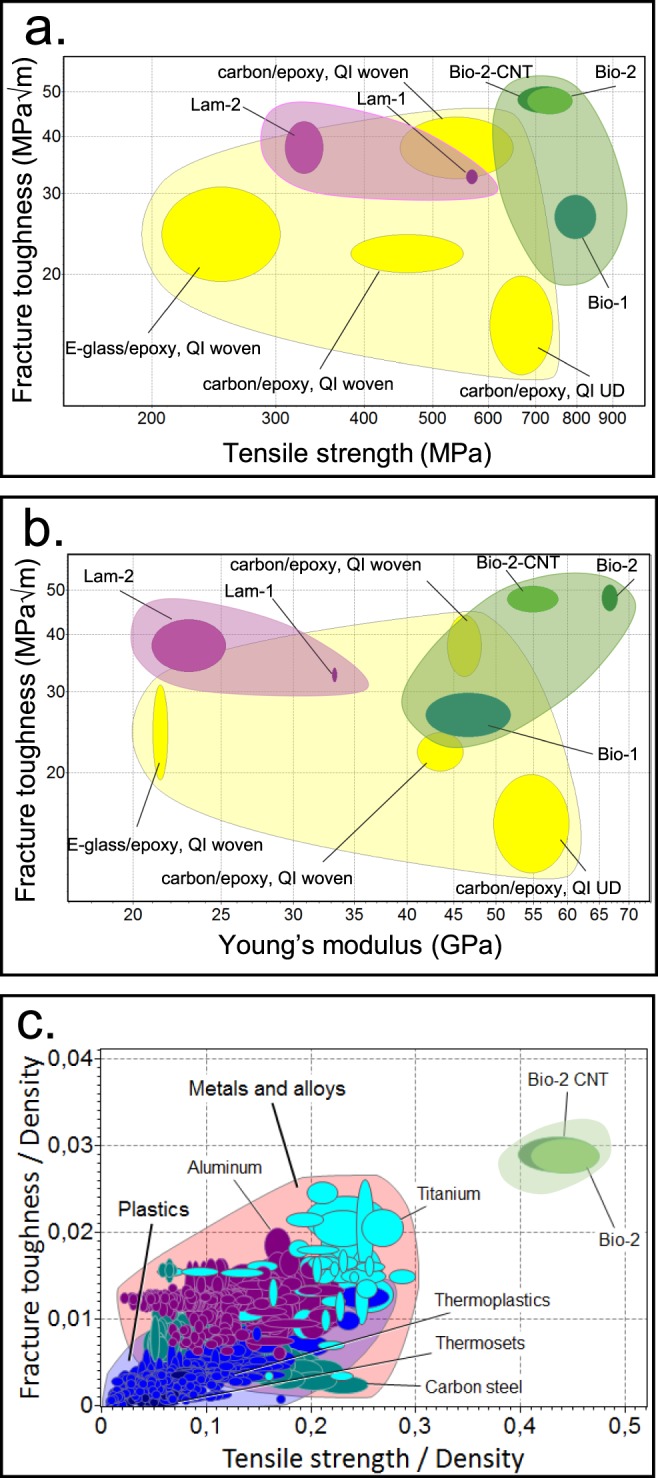
Comparison with composite laminates and other structural materials. (**a**,**b)** Ashby plots showing a direct comparison among the proposed bioinspired solutions (Bio-2 and Bio-2-CNT), the comparative laminates (Lam-2 and Lam-1^45^), the previous design solution Bio-1^45^, and other currently adopted structural composites made of GF and CF, having similar fiber content, 50% vol., and similar quasi-isotropic (QI) lay-ups with fibers orientated at (0/90/+45/−45). (**a)** Fracture toughness vs Tensile strength. (**b)** Fracture toughness vs Young’s Modulus. Enhanced mechanical properties are achieved through a biomimetic design: the new solutions (Bio-2 and Bio-2-CNT) show noticeable higher performance with respect to the previous design, Bio-1, presented in^45^ and with respect to the classic laminates, Lam-2 and Lam-1^45^, fabricated for comparative aims. An evident increase in toughness and strength is also achieved with respect to different commercially used laminates, having the same fiber volume fraction. (**c)** Comparison between the proposed bioinspired solutions (Bio-2 and Bio-2-CNT) and other conventional structural material, e.g. Metals, Alloys, and Plastics in terms of specific fracture toughness vs. specific tensile strength. Unity measures: [MPa·m^3/2^/kg] and [MPa·m^3^/kg] on the vertical and horizontal axis, respectively.

Toughness is also considerably higher than the corresponding value of similar carbon-glass-epoxy FRCs (data taken from CES EduPack, Granta Design Limited, 2018) with a similar fiber content (~50% vol.) and comparable quasi-isotropic stacking sequences (e.g. containing 0°, 90°, and ±45°-oriented fibers). The Ashby plots in Fig. 3 reveal the exceptional toughness of the newly designed materials (Bio-2 and Bio2-CNT) and a great balance with stiffness and strength, when compared to other quasi-isotropic laminates. They also have better performance compared to quasi-isotropic laminates, made of only carbon fibers, which generally have higher performance and costs than fiberglass. The proposed bioinspired architectures reach an optimal toughness-stiffness and toughness-strength tradeoff, overcoming a typical material-design issue^1^, and providing a strategic alternative to currently adopted composite solutions. Moreover, considering the advantage given by the low weight, this design may also offer a better choice, in terms of specific toughness, compared to traditional materials, such as metals and polymers (Fig. 3c).

### Analytical approach

**Figure 4:**
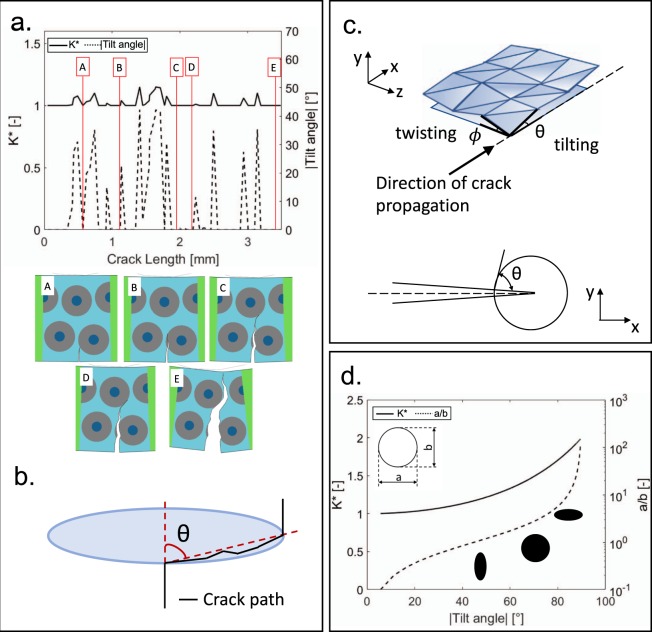
Effect of the reinforcement. (**a)** Graph showing the trend of titling angle (as absolute value) and the trend of K* with respect to the crack length: each peak is correlated with the corresponding snapshot showing the crack propagation in the numerical model. Peaks of K* are associated to the continuous deviation of the crack during the deflection around the osteon cement line. (**c)** Definition of the tilting angle, θ. (**c)** Schematic of crack deflection: in-plane deflection (tilting) and out-of-plane deflection (twisting). Definition of tilting angle between the direction of crack propagation and the direction of deviation. (**d)** Toughening effect of the reinforcement shape: K* increases as a function of the tilting angle. Beneficial effect of elliptical reinforcement (with the main axis parallel to the direction of crack propagation) on the overall fracture toughness.

To further investigate the beneficial effect of this bioinspired design on the fracture response, we follow an analytical approach to describe the mechanics of crack propagation and growth resistance in this complex structure. This approach allows us to elucidate how the structural features (osteon, cements lines) affect the crack path and the fracture response, and provides details for crack propagation control. Resistance to crack growth can be increased by engineering the material architecture so as to reduce the stress field at the tip, e.g. by crack deflection, or tip shielding. Crack tip deflection occurs when planes of weakness are introduced in a material. The forces necessary for crack deflection can be calculated by means of a Griffith-type energy balance to evaluate the increase in applied stress-intensity factor needed for propagation at either a tilt angle, *θ*, or a twist angle, *ϕ*, with respect to the original plane (Fig. 4c). The stress-intensity factors, *K*(*θ*) and *K*(*ϕ*), associated to the tilting and twisting conditions are given by the following equations^55^:

1 $$K(\theta )={K}_{IC}se{c}^{2}(\theta /2)$$

2 $$K(\varphi )={K}_{IC}se{c}^{2}(\varphi )$$

To reveal the effect of the bioinspired arrangement, we use the finite element model that reproduces the transversal flexural loading (Fig. 1d). Being a 2D-model, we can only focus on the tilting condition. The tilt angle in the three-point bending simulation can be stepwise measured, along the crack propagation path, with the aid of the software ImageJ^56^. Being *K*_*IC*_ a material constant, Equation (1) can be rearranged as follows:

3 $${K}^{\ast }=\frac{K({\rm{\theta }})}{{K}_{Ic}}=se{c}^{2}(\theta /2)$$

Fig. 4a shows how K* varies during the crack propagation, being affected by the continuous deviations along the cement sheath. We follow the propagation path as in Fig. 4a, where crack initiation occurs in the interstitial matrix. When encountering the osteon, the crack first penetrates the outer layer, then circumvents the osteon, entering again the matrix. Afterward, it experiences another deflection, kinking around another osteon before the final failure. On the graph in Fig. 4a we can observe the peaks corresponding to all the deviations that contribute to a progressive energy dissipation, preventing a sudden rupture.

As Faber and Evans showed^57^, the increase in fracture toughness for materials with inclusions depends on the particle shape and content. To study the effect of the osteon shape, we consider a simplified model of a crack growing into a matrix and reaching an osteon, where the tilt angle is described as in Fig. 4b. By setting “a” and “b” as the horizontal and vertical osteon axes, respectively, we can quantify the osteon shape effect on K*. As an example, increasing the a/b ratio from 1 to 1.33 boosts K* by 14%. Fig. 4d illustrates how toughening is affected by the reinforcement shape, endorsing the beneficial effect of an elliptical-shaped osteon (with the main axis perpendicular to the direction of crack propagation) on the overall fracture toughness. This beneficial effect has also been experimentally observed by the authors in a previous work^46^.

By comparing Eqs (1) and (2) and considering two equivalent angles (*θ* = *ϕ*), we also notice how the crack deflection owing to twisting is much more effective, in terms of increase in toughness, than crack deflection owing to tilting. This opens new venues for future designs, where the architecture can be engineered to offer both the titling and twisting mechanisms, and the reinforcement geometry fine-tuned to promote superior energy dissipation. Future works could include the adoption of an elliptical osteon reinforcement with multiple orientations, to favor deviations along different planes. Going forward, the design could be further improved also leveraging optimization techniques.

## Remarks

In this paper, we proposed a novel biomimetic paradigm, rooted into characteristic design motifs (e.g. fibrous, layered, tubular, and overlapping) for designing and fabricating advanced structural materials. We envisioned and demonstrated the effectiveness of a bioinspired design, implemented into a fiber-reinforced composite, to increase the fracture toughness with respect to currently adopted structural materials, and balance with stiffness and strength. This goal is attained by synergistically activating the characteristic toughening mechanisms of fiber-reinforced composites and those promoted by the bioinspired architecture. By mimicking the main structural features of bone microstructure, e.g. the osteons, we could implement the characteristic toughening mechanisms, which are considered the hallmark of bone enhanced fracture toughness, boosting the fracture performance by 86% and 26%, compared to a previous design and to classic laminated composites, respectively, and offering an optimal tradeoff with stiffness and strength. XFEM simulations have contributed to elucidate the mechanisms of crack deviation in the biomimetic architecture and to gain insights into the role of the structural features and their arrangement on the overall mechanical performance and failure process. Thereafter, we demonstrated, using a joint analytical-numerical approach, how to control and direct the crack, by tailoring the material architecture, providing new routes for future design improvements.

Reaching a remarkable strength-toughness balance combined with a low weight, this material and its design strategy has the potential to significantly improve the safety of advanced structures and components, with a profound impact in both academic and industrial fields. The demonstrated superior fracture toughness represents a fundamental leap for structural materials, and we expect further progress in terms of properties and weight reduction by implementing additional strategies, from accurate selection of building blocks (e.g. carbon and boron fibers), to fine-tuning the reinforcement geometry (e.g. implementing elliptical shapes) and promoting multi-dimensional hierarchical approaches (e.g. further investigating the effect of nano-reinforcement).

## Methods

### Design and Materials

Three types of composite materials have been designed and manufactured:

1. the bioinspired material, named Bio-2
2. the bioinspired material, named Bio-2-CNT
3. the classic laminate, named Lam-2

The design and characteristics of each composite type are given in Table S1, Supplementary Information.

### Manufacturing

Multiple plates for each composite type are produced (bioinspired plates, bioinspired plates with CNTs, comparative conventional composite plates), to allow a comprehensive characterization of each design solution and a proper comparison of the performance.

#### Bio-2

To manufacture Bio-2 and Bio-2-CNT we develop an ad hoc technique based on hand preforming and VARTM. We build a frame, to facilitate the tube placement and alignment. Then we build a rigid mold, in which the fabrics and tubes are attached together using double sided tape, to ensure the compaction and achieve an osteon volume fraction of 60%. To inject the resin, we adopt an injection procedure based on the VARTM technique, which could facilitate the impregnation of a complex system with a good quality, such as the bioinspired design. The main complexity derives from: a) the CF-tubes, which are available as dry fabric and not prepreg, b) the uneven surface caused by the osteon distribution, and c) the impregnation anisotropy. The resin is mixed with the hardener with a weight ratio of 10:3 and processed in a vacuum planetary mixing machine, to ensure a uniform mixture of the components and to extract the air inclusion. During the impregnation, the mold is placed vertically and a vacuum of 850 kPa is imposed, to prevent the resin passing through the reinforcement without properly impregnating it.

#### Bio-2-CNT

To add CNTs to the previous bioinspired material structure, Bio-2, and create a further hierarchical level, a small quantity (0.1%wt.) of multi-walled carbon nanotubes (MWCNT) is dispersed in the epoxy by ultrasound sonication for 20 mins (to allow an even distribution), then the hardener is added. For manufacturing, the same lamination, injection, and curing setup, adopted for Bio-2, is used. The amount of CNTs is chosen so as to ensure a proper resin flow and a good impregnation. A larger amount, indeed, would increase the resin viscosity resulting in material defects (e.g. resin starving, debonding).

#### Lam-2

Lam-2 is designed to have the same thickness and constituent materials of the bioinspired designs.

Consequently, the fiber volume fraction of all the three materials, developed in this work, is about 50% and the fibrous constituents of the material are about 50% CF and 50% GF. The types of resin and fiber and the amount of fibers placed in a specific orientation are the same for all the material solutions. In particular, the manufactured classic laminate, Lam-2, has the following stacking sequence: [GF(0°–90°)_2_, CF(±45°)_2_, GF(0°), CF(±45°)_2_, GF(90°)_2_]_s_. Further details are indicated in Table S1, Supplementary Information.

By comparing the impregnation process of the bioinspired structure, Bio-2, with that of the classic laminate, Lam-2, we notice that the tubular features of the bioinspired topology are causing an uneven impregnation front, with a higher impregnation speed in the channels between the osteons. We believe that this behavior is due to the difference in fiber volume fraction between the inner and outer regions of the tubes. Indeed, a macro resin flow rapidly passes in-between the tubes, while the inner parts of the tubes are slowly impregnated, by capillarity. It is therefore expected that a higher dual-scale flow behavior could lead to high porosity volumes in the tubes owing to resin starving. This phenomenon is emphasized for the Bio-2-CNT composite plates, by the increase of viscosity caused by the addition of CNTs to the resin.

To assess the quality of the plates, and test what stated above, we cut some samples, polish the surfaces and observe them using an optical microscope. As it is shown in Fig. S1a, Supplementary Information it is possible to find porosities in the center of the tubes, caused by resin starving. In Fig. S1b, Supplementary Information a comparison between the two impregnation fronts, for the Bio-2 and Lam-2 case study, is shown.

After setting up all the parameters and check the quality of the plates, we manufacture 5 plates from which we cut the samples, using the Waterjet technology, to ensure a proper finishing. We only test the samples obtained from the plates with a good finishing and we exclude from the study the plates showing barely visible manufacturing-induced defects.

### Mechanical testing

All the tests are performed on all the composite types in both longitudinal and transversal orientations. The orientation is defined with respect to the main reinforcement feature (e.g. the osteon-like tube). All the details regarding the mechanical testing performed on the composites are indicated in Table S2, Supplementary Information whereas the outcome of all the testing are given in Table S3, Supplementary Information. Besides the tests on composites, we also perform tensile tests on epoxy samples and on samples made of epoxy resin doped with CNTs, to evaluate the effect of the CNTs on the pure resin. The tests are performed following the ASTM D638-10^58^. It is necessary to state that, owing to the use of the extensometer, 2 out of 3 epoxy/CNTs samples are deformed by its weight, influencing the results. Although the data of maximum stress and Young modulus are considered valid, we decide not to consider valid the data of elongation at breakage and toughness modulus for the samples affected by the extensometer. From these tests, we do not notice a significant effect of the CNTs, as indicated by the results in Table S4, Supplementary Information.

#### Tensile tests

For the tensile tests, we follow the standard ASTM D3039/D3039M-08^59^. We adopt rectangular samples. However, some dimensions, such as the thickness and width, are slightly modified compared to those recommended by the standard to fit our plate dimensions. For instance, the thickness is fixed by that of the manufactured plates, whereas the width, instead, is increased to 20 mm for the longitudinal samples so as to include in each specimen a more statistically relevant quantity of tubes. The specimens are endowed with adhesively bonded tabs at both ends, to ensure a correct load transfer through the grips, avoiding stress concentration and misalignment owing to the grip pressure (equal to 15 MPa). Tabs are bonded with an epoxy adhesive glue (Araldite DP490).

#### Compression tests

For compression tests, we follow the standard ASTM D3410/D3410M-03^60^. Samples are cut in rectangular shape and tabs are glued before testing.

#### Three-point-bending tests

For three-point-bending tests, we follow the European standard, UNI EN ISO 14125^61^.

#### Translaminar fracture toughness tests

For the translaminar fracture toughness tests we follow the standard ASTM E1922-04^62^, which describes the procedure for the determination of translaminar fracture toughness, *K*_*TL*_, for laminated and pultruded polymer matrix composite materials, using test results from monotonically loaded notched specimens. Additionally, this type of test allows us to investigate how the fracture propagates in both the bioinspired composites (Bio-2 and Bio-2-CNT) and the classic laminate (Lam-2), allowing a final comparison on this fundamental mechanical characteristic. The specimen geometry for this test is the eccentrically single edge notch tension specimen, ESE(T), loaded in mode I. We use waterjet to cut the main rectangular sample shape and a diamond impregnated copper slitting saw to cut the notch. From this test is possible to quantify the translaminar fracture toughness (*K*_*TL*_) of the materials and to analyze how the fracture propagates in the materials, underlining the different failure modes. A displacement gage is used to measure the displacement at the notch mouth during loading. The gage is attached to the notch edges using adhesively bonded knife-edges.

### Numerical model

The model is based on the XFEM, implemented in Abaqus 6.14. XFEM, initially developed by Belytschko and Black^63^, and recently implemented into commercial FE-codes, allows the simulation of discontinuities (e.g. crack propagation) in an element, by enriching the degrees of freedom with special displacement functions. Contrarily to the classic FEM, XFEM does not require remeshing in the crack tip region, being mesh independent. Moreover, the crack position may or may not be pre-determined. In the latter case, XFEM locates the possible crack initiation position by detecting the element that corresponds to the critical state, identified by the adopted damage initiation criterion (e.g. stress- or strain-base criterion).

We perform quasi-static simulations. Our XFEM-based modeling framework is based on the cohesive segment approach, which uses the traction-separation constitutive laws. The mechanical behavior is characterized by three regions: i) linear elastic, ii) damage initiation, and iii) damage evolution. The elastic properties define the initial tract, while damage initiation in the XFEM enriched region is set by the critical maximum principal stress criterion (MAXPS), similarly to other previous studies on fiber-composites^47,64,65^. According to MAXPS, initiation occurs when the maximum principal stress, *σ*_*n*_ reaches a critical value, ${\sigma }_{{\max }}^{0}$ (i.e. *f* = *1* in Eq. (4)).

4 $$f=\frac{{\sigma }_{n}}{{\sigma }_{{\max }}^{0}}$$

Crack propagation and how the material cohesive stiffness degradation occurs are set by the damage evolution properties, which are described by energy- or displacement-based criteria. To describe the damage evolution, we adopt a displacement-based criterion.

Our model replicates the transversal three-point bending case study (Fig. S2, Supplementary Information). The geometry and the dimensions correspond to those of the experimental sample, designed according to the standard UNI-EN ISO 14125^61^. Non-specimen parts (i.e. loading member and rigid supports) are modeled as analytical rigid components. The displacement is applied to the loading member, while the rotation and displacement of the rigid supports are constrained in all directions. Surface contact between the specimen and the loading and support members is set to occur in a tangential behavior using a penalty formulation and a friction coefficient of 0.001. To reduce the computational effort, we model the topological pattern only in the central region, whereas in the other region we adopt a homogenous equivalent material. The definition of the subregions is shown in Fig. S3, Supplementary Information. A local enrichment is assigned to the central region and no initial crack location is defined. The model consists of 6193 four-node bilinear plane stress quadrilateral elements with reduced integration (type CPS4R). The central region includes elements with 0.06 mm size, while the homogeneous regions include elements of 2 mm. To allow a smoother mesh transition, we also define an intermediate region.

The material properties adopted in the XFEM model are listed in Tables S5 and S6, Supplementary Information.

## Supplementary information

Bone-inspired enhanced fracture toughness of de novo fiber reinforced composites
