# Supplementary material for: Bone-inspired enhanced fracture toughness of de novo fiber reinforced composites
Source: Sci Rep. 2019 Feb 28;9:3142. doi: 10.1038/s41598-019-39030-7 (PMC6395722; doi:10.1038/s41598-019-39030-7)
Supplement: Supplementary file 1 — Bone-inspired enhanced fracture toughness of de novo fiber reinforced composites [file 41598_2019_39030_MOESM1_ESM.docx]

**Supplementary Information**

**Bone-inspired enhanced fracture toughness of de novo fiber reinforced composites**

Flavia Libonati^1^, Andre E. Vellwock^1^, Francesco Ielmini^1^, Dilmurat Abliz^2^, Gerhard Ziegmann^2^, and Laura Vergani*^1^*

*^1^Department of Mechanical Engineering, Politecnico di Milano, via G. La Masa 1, 20156 Milano, Italy*

*^2^Department of Polymer Materials and Plastics Engineering, Clausthal University of Technology, Clausthal-Zellerfeld, Germany*

*Corresponding author, email address: [flavia.libonati@polimi.it](mailto:flavia.libonati@polimi.it)

**This file includes:**

Design and Materials

Manufacturing

Mechanical Testing

Numerical Model

Figures S1, S2, and S3

Tables S1 to S6

**Design and materials**

**Table S1.** Design and characteristics of each composite type.

**Manufacturing**

1. ** b.**
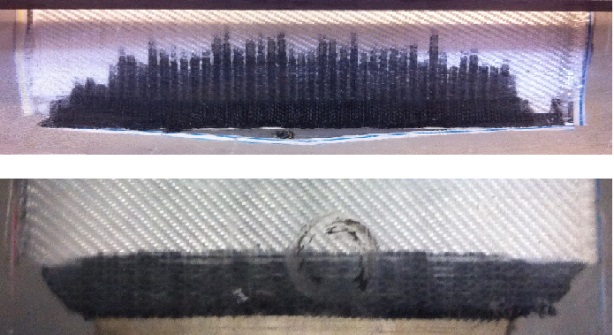


**Figure S1.** **a)** Impregnation-induced defects (picture from SEM, scale bar 200 μm). **b)** Impregnation front for Bio-2 (top inset) and Lam-2 (bottom inset).

**Mechanical testing**

**Table S2.** Details about testing setup and parameters, sample geometry and standard followed.

**Table S3.** Results of the whole experimental campaign carried out to perform a complete characterization of different design solutions: Bio-2, Bio-2-CNT, and Lam-2.

**Table S4.** Comparison between the tensile properties of pure epoxy samples and those of epoxy+CNTs samples.

**Numerical model**


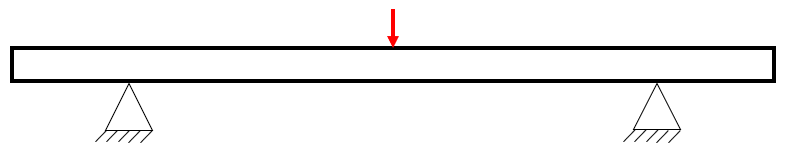


**Figure S2.** Schematics of loading and boundary conditions.

The XFEM simulations are based on the cohesive segment approach, which uses the traction-separation constitutive laws. The mechanical behavior is characterized by three traits:

1. linear elastic
2. damage initiation
3. damage evolution

The model, aiming at simulating the three-point bending configuration (**Figure S2**), is divided into different subregions, as schematically represented in **Figure S3**: i) internal lamellae, ii) cement line, iii) interstitial matrix, and iv) homogeneous. The non-central region, with a coarser mesh, is modeled as homogeneous, with material properties determined from experiments and summarized in **Table S3**. For the central area, the material properties of each subregion are assigned according to **Table S5** and **Table S6**.

The cement line region is made by CF/epoxy and the material properties are taken from a previous study^1^, where the co-authors built a submodel of the [±45°] fabric using Texgen 3.9, and Abaqus. The internal lamellae subregion, inside the cement line, is modeled as a UDGF/epoxy, where the fibers are parallel to the z-direction (i.e. oriented orthogonally to the xy model plane). Therefore, in the 2D-model, this subregion can be considered isotropic in-plane and has the properties of transversal laminates, experimentally determined in a previous study^2^. To reduce the computational cost and allow a more regular mesh, the GF outer layers, the UDGF inter-osteons layers and the resin-only regions are modeled as a single subregion, assuming the fibers aligned in x-direction. The mechanical parameters are those of longitudinal laminates, experimentally determined in a previous study made by the authors^2^. The damage parameters are set only for the enriched region (i.e. the middle one).

As critical stress for damage initiation, we assume the maximum stress experimentally determined by the authors in a previous study on GFRP^3^, for both the internal lamellae and the interstitial matrix. For the cement line, instead, we use the tensile strength of [±45°] carbon/epoxy included in the datasheet supplied by the producer. For each subregion of the enriched area (i.e. middle part of the sample), the displacement at fracture is calculated using the characteristic length (0.085 mm), which is the diagonal dimension of a rectangular element (0.06 mm) of that region, and the corresponding strain at fracture.

**Figure S3.** a) Schematic of the model: the bone-like topology is implemented only in the central region, whereas the rest is simplified as homogeneous. b) Magnification of the central region and definition of four different subregions. The top, bottom, and internal GF[90°] layers impregnated into epoxy are simplified as a unique region named interstitial matrix. c) Mesh transition. d) Central region with finer mesh.

**Table S5.** Material properties adopted in the numerical model.

| **Region** | **Dimension** | **Material** | **Young modulus [MPa]** | **Poisson ratio [-]** | **Maximum Principal Stress [MPa]** | **Displacement at fracture [mm]** |
| --- | --- | --- | --- | --- | --- | --- |
| **Internal lamellae** | 0.4 mm (diameter) | UDGF/epoxy | 9718^2^ | 0.077^2^ | 59^2^ | 0.0011^2^ |
| **Cement line** | 1.2 mm (External diameter) | CF/epoxy | **Table S6** | **Table S6** | 600* | 0.0007* |
| **Interstitial matrix** | - | GF/epoxy | 37673^2^ | 0.3^2^ | 388^2^ | 0.0034^2^ |
| **Homogeneous** | - | - | 20034 | 0.2 | - | - |

*from the datasheet supplied by the producer.

**Table S6.** Materials properties of the CF/epoxy region from Vellwock et al.^1^

| **Material** | **E1 [MPa]** | **E2, E3 [MPa]** | **ν12, v13 [-]** | **v23 [-]** | **G12, G13 [MPa]** | **G23 [MPa]** |
| --- | --- | --- | --- | --- | --- | --- |
| **CF/epoxy** | 10000 | 50000 | 0.11 | 0.54 | 3700 | 2500 |

**References**

1 Vellwock, A. E., Vergani, L. & Libonati, F. A multiscale XFEM approach to investigate the fracture behavior of bio-inspired composite materials. *Composites Part B: Engineering* **141**, 258-264, (2018).

2 Libonati, F. & Vergani, L. Damage assessment of composite materials by means of thermographic analyses. *Composites Part B: Engineering* **50**, 82-90, (2013).

3 Libonati, F., Colombo, C. & Vergani, L. Design and characterization of a biomimetic composite inspired to human bone. *Fatigue & Fracture of Engineering Materials & Structures* **37**, 772-781, (2014).
